# Utility of micro-TESE in the most severe cases of non-obstructive azoospermia

**DOI:** 10.1080/03009734.2020.1737600

**Published:** 2020-04-01

**Authors:** Göran Westlander

**Affiliations:** Livio Fertility Center, Göteborg, Sweden

**Keywords:** Azoospermia, *in vitro* fertilization, male infertility, micro-TESE

## Abstract

The use of intracytoplasmic sperm injection (ICSI) has been a major breakthrough in the treatment of male infertility. Even patients with non-obstructive azoospermia (NOA) may benefit from the ICSI technique to father a child as long as spermatogenesis is present. There are several techniques to recover testicular sperm in patients with NOA. However, retrieval of spermatozoa is unfortunately still only successful in a subset of patients with NOA, and the most superior sperm retrieval method is still under debate. A more recent technique, microdissection testicular sperm extraction (MD-TESE) with an operative microscope collecting larger and more opaque seminiferous tubules, is a non-blind sperm retrieval technique with theoretical benefits. The MD-TESE procedure seems to be feasible, effective, and safe in NOA patients but also more technically demanding and time-consuming compared with conventional blind techniques. In the present report, we describe our clinical experience and results from our first 159 MD-TESE procedures. The probability to retrieve sperm with the MD-TESE technique is high in NOA cases where earlier sperm retrieval with blind methods such as needle aspiration, percutaneous needle biopsy, or conventional TESE has failed.

## Introduction

The introduction of intracytoplasmic sperm injection (ICSI) in 1992 revolutionized the treatment of male infertility (1). The most severe form of male infertility is non-obstructive azoospermia (NOA) where spermatogenesis is impaired or totally absent. Azoospermia is defined by the complete absence of spermatozoa in at least two semen analyses and is present in approximately 1% of adult men. If testicular spermatozoa can be retrieved, men with NOA can achieve biological fatherhood by means of ICSI (2).

**Figure 1.**
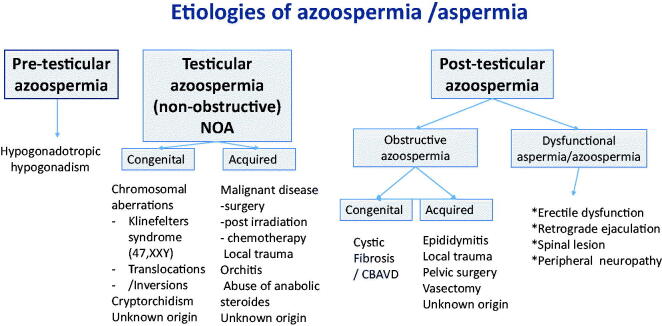
Aetiologies of azoospermia/aspermia.

The aetiology of azoospermia is divided into three groups: pre-testicular, testicular (non-obstructive), and post-testicular (Figure 1). Pre-testicular cases can often be treated successfully with hormones. Men with post-testicular (obstructive) azoospermia are usually normogonadotropic with normal spermatogenesis. Then, spermatozoa can easily be recovered for ICSI by percutaneous epididymal sperm aspiration (PESA) (3) or testicular sperm aspiration (TESA) (4,5).

Men with testicular azoospermia (NOA) have impaired spermatogenesis, and surgical testicular procedures are required to retrieve sperm. There are several techniques available, such as TESA (4,5), percutaneous testicular biopsies (6), and open biopsies (conventional testicular sperm extraction, cTESE) (2) (Supplementary Figure 1; available online). The overall success rate of retrieving sperm with these techniques varies between 25% and 50% in most reports (2,4–6).

A more recent technique, microdissection testicular sperm extraction (MD-TESE), has been shown to achieve significantly better sperm recovery rates compared with cTESE (7,8). MD-TESE is a ‘non-blind’ technique where seminiferous tubules are directly examined throughout the testis using an operative microscope (Supplementary Figure 1; available online). This is in contrast to TESA, percutaneous testicular biopsies, and cTESE, which all are blind techniques. The MD-TESE procedure seems to be feasible and safe in NOA patients but also more technically demanding and time-consuming compared with cTESE (7,9).

In Sweden, TESA and percutaneous biopsies are traditionally the most common methods to retrieve sperm for ICSI in patients with NOA. These procedures are carried out in most of the Swedish fertility centres because of their simplicity and low costs. Sperm retrieval rates (SRR) with TESA vary, but, with an optimal technique, SRR have been reported as high as 35% (4,10). In 2013 Livio Fertility Center in Göteborg was the first Swedish IVF clinic to introduce MD-TESE, but recently additional centres have introduced it as well. However, due to the lack of large randomized controlled trials the superiority of this new technique is still questioned.

The aim of this report is to describe our own clinical experience and results with MD-TESE and discuss how this technique is best administered and performed in relation to hormonal treatment of and egg recovery from the female partner.

### Microdissection TESE, surgical procedure

Procedures are preferably performed under local anaesthesia. A midline incision is made into the scrotum, and the tunica vaginalis is opened. The testis covered with the tunica albuginea is visualized, and the testis is opened widely in an equatorial plane along the mid-portion. This allows for wide exposure of seminiferous tubules in a physiological approach that follows intratesticular blood flow (11). The remainder of the procedure is performed under an operative microscope at ×20–25 magnification. Small samples are excised from the tubules. Larger and more opaque tubules are more likely to contain sperm (Supplementary Figure 2; available online). Up to 15 biopsies on each side are taken, but this can vary due to testicular size and tubular status. The procedure is terminated when all areas of the visualized parenchyma have been examined under the microscope or when further dissection is thought likely to jeopardize the testicular blood supply.

### Biological search for spermatozoa

Fragments of testicular tissue are placed in IVF medium in sterile Petri dishes and minced using micro-scissors or scalpels. Fractions are put in droplets of fertilization medium under oil, and thereafter well-trained embryologists begin their search for spermatozoa.

### Preoperative preparation

Before surgery, at least two semen analyses have been performed to confirm azoospermia. Moreover, serum-FSH, LH, testosterone and SHBG concentrations have been analyzed to distinguish between obstructive and non-obstructive azoospermia and to exclude hypogonadism. A physical investigation has been performed measuring testicular volume with an orchidometer, and testicular ultrasonography to exclude scrotal abnormalities such as varicocele, epididymal/testicular cysts, hydrocele, and testicular tumours. Finally, a karyotype has been analyzed in most NOA men, and screening for Y-chromosomal microdeletions carried out as well.

### Hormonal treatment

Men with NOA suffer from testicular insufficiency. Serum FSH concentrations are most often elevated, and serum testosterone is borderline to low. It is controversial whether low testosterone concentrations predict the success of MD-TESE. Medical therapies that increase serum testosterone might also increase the intratesticular testosterone levels, which might be beneficial for spermatogenesis (12). Drugs often used are aromatase inhibitors and human chorionic gonadotrophin (HCG) (11).

### Clinical predictors of positive sperm retrieval

At present, there is only one good clinical predictor of negative sperm retrieval in men with NOA. If screening for Y chromosome microdeletions reveals complete deletion in the AZF-a or AZF-b areas, the likelihood of finding sperm with any surgical sperm recovery technique is negligible (13). A sufficient number of sperm aimed for ICSI can be surgically recovered independently of small testicular volumes, high FSH, or low testosterone concentrations (14,15).

### Sperm retrieval rate according to testicular histology

The most common histological conditions seen in men with NOA are hypospermatogenesis, Sertoli cell-only syndrome (SCOS), maturation arrest, and atrophy (7). Hypospermatogenesis is characterized by a reduced number of germ cells, and all stages of spermatogenesis are present. These men have the best probability for a successful sperm recovery by surgical retrieval. MD-TESE, cTESE, as well as sperm aspiration techniques (TESA, FNA) all have acceptable positive SRR (4,5,7).

SCOS is associated with the absence of germ cells, with normal Sertoli cells exclusively lining the seminiferous tubules. However, focal areas with intact tubules can be found especially with the ‘non-blind’ MD-TESE technique. Men with SCOS have lower SRR compared with other histological conditions with SRR ranging from 22% to 44.5% (7,15). However, in the current literature, MD-TESE seems more beneficial with successful sperm retrieval in histological patterns of patchy spermatogenesis such as SCOS. In patients with uniform histological patterns such as maturation arrest, the outcome of MD-TESE seems less favourable compared with other retrieval techniques (7).

### Clinical routines and results

With the exception of complete Y-chromosomal microdeletions in AZF-a and AZF-b regions, no secure clinical predictors of sperm retrieval have been demonstrated (16). Therefore, most patients with NOA in our fertility centre are offered operative surgical sperm retrieval. First-line treatment is usually a diagnostic TESA where multiple testicular samples of testicular tissue are collected with a 19-gauge butterfly needle. In case of insufficient amounts of recovered tissue, procedures can be complemented with percutaneous needle biopsies where biopsy cylinders are harvested in different regions of the anterior part of the testis. Procedures are performed under local anaesthesia, and SRR in our centre are up to 34% in patients with NOA (5,10). Spermatozoa are cryopreserved if possible. If sperm are retrieved, a subsequent IVF cycle is offered with a repeated TESA procedure, alternatively with thawed testicular sperm. If no sperm are found, patients are offered MD-TESE in parallel with hormonal stimulation and egg recovery from the female partner.

The results after our first 159 MD-TESE procedures are shown in Table 1. Twelve repeated cycles were excluded. In one out of 12 repeated procedures, sperm were not retrieved. One patient had two repeated procedures. Altogether the 12 repeated cycles resulted in another five deliveries. Most cases had undergone a previous unsuccessful sperm retrieval such as TESA or cTESE. Sperm sufficient for ICSI were identified in 51% of cases after MD-TESE. Seventy-six percent of cases where ICSI was performed resulted in fertilization and development of at least one good-quality embryo and embryo transfer. A maximum of two embryos were transferred at the cleavage stage and only one embryo at the blastocyst stage. Sixty-two first cycle embryo transfers resulted in 22 (35%) ongoing pregnancies/deliveries. In addition, from the 81 cases with positive sperm retrievals, 14 more ongoing pregnancies/deliveries have been reported from subsequent IVF cycles using thawed testicular sperm or thawed vitrified blastocyst embryos.

**Table 1.** All non-obstructive azoospermia patients.

	Number	Per cent
Number of patients	159	
Number of MD-TESE performed	171	
Men with retrieved sperm first procedure	81	51 (81/159)
Cycles leading to embryo transfer	62	77 (62/81)
Pregnancies per embryo transfer	30	48 (30/62)
Ongoing pregnancies/deliveries per embryo transfer	22	35 (22/62)
Cumulative number of ongoing pregnancies/deliveries after MD-TESE procedures	36	21 (36/171)

We do not consider MD-TESE to be the first line of treatment, with the exception of very small atrophic testes such as in Klinefelter syndrome. Up to one-third of the patients with NOA in our fertility centre have sperm sufficient for ICSI with needle aspiration techniques. Starting work-up procedures in NOA cases with a diagnostic TESA will reduce the number of requested procedures of MD-TESE, which is more invasive, time-consuming, and expensive compared with the needle aspiration techniques. TESA can easily be repeated in a subsequent IVF cycle if sperm is retrieved and if cryopreservation of testicular sperm is not possible. However, a subsequent MD-TESE procedure in case of negative sperm retrieval after TESA is well motivated, as more than half of NOA patients who fail with diagnostic TESA/cTESE are able to have sperm retrieval with a dedicated MD-TESE (10,15).

## Discussion

At present, there are no doubts that MD-TESE has theoretical benefits compared with the ‘blind’ recovery techniques such as TESA, percutaneous biopsies, and cTESE. A tendency towards a higher SRR using MD-TESE has been reported, but good clinical randomized studies are still lacking (7). In our fertility centre more than 170 MD-TESE procedures have been performed, most of them with a previous ineffective TESA procedure. Sperm sufficient for ICSI were found in more than 50% of the cases. In 76% of these cases, fertilization and embryo development were normal, resulting in embryo transfer. Ongoing pregnancy/delivery rate per embryo transfer was 35% when only the first treatment MD-TESE/IVF cycles were included. The overall encouraging results, highly comparable with our other conventional IVF results, might be explained by the relatively low age of the female partners. In most IVF cycles, the female age was <40 years in combination with at least a normal egg reserve. Two embryos were transferred in a majority of cases, but only three twin pregnancies have been reported. This is most likely explained by the lower implantation rate when using testicular sperm for ICSI in men with NOA (17).

Our reported results are similar to those of a systematic review (8), where SRR with MD-TESE technique in eight different studies ranged between 43% and 63%. A recently published meta-analysis reported SRR with MD-TESE in 15 studies between 2000 and 2019. The pooled SRR of the series was 45% (95% CI 39–54%). Compared with needle aspiration techniques (TESA), MD-TESE seemed to be superior (18).

Klinefelter syndrome (KS) is the most frequent group of chromosomal abnormalities in men with NOA, with a prevalence ranging from 1:500 to 1:700 in newborn males. Because of the genetic alteration – men with KS represent a group with one extra X chromosome added to the male karyotype, 46,XY – there is progressive testicular damage leading to impaired or absent spermatogenesis. A meta-analysis from 2017 with 37 studies included (19) reported an overall SRR of about 40%. Among our 159 patients with MD-TESE procedures, 19 were diagnosed with non-mosaic KS. Our SRR (37%) was similar to previous reports. Interestingly, in cases where sperm were retrieved, 86% resulted in embryo transfer, and ongoing pregnancy/delivery rates per embryo transfer were high (67%). In subjects with KS, we can report a final live birth rate (LBR) of 32% (6/19) for the couples who initiated IVF in combination with MD-TESE (Table 2). This is higher compared with the meta-analysis from Corona et al. (19) reporting an overall LBR of 16% per couple who initiated the assisted reproductive techniques. However, only 19 subjects were enrolled in our report. All children born were healthy with a normal karyotype, which is in line with other reports showing a risk of chromosomal abnormalities similar to that reported in subjects without KS. At present, many published studies have shown high SRR in men with KS, and conception appears safe (19). Unfortunately, many fertility doctors, not aware of the treatment possibilities for KS men, recommend treatment with donor sperm instead of referring couples to centres performing MD-TESE and IVF.

**Table 2.** MD-TESE in patients with Klinefelter syndrome.

	Number	Per cent
Number of patients	19	
Number of procedures	20	
Men with retrieved sperm first procedure	7	37 (7/19)
Cycles leading to embryo transfer	6	86 (6/7)
Pregnancies per embryo transfer	6	100 (6/6)
Ongoing pregnancies/deliveries per embryo transfer	4	67 (4/6)
Cumulative number of ongoing pregnancies/deliveries after MD-TESE procedures	6	30 (6/20)

Policies differ between clinics in the treatment of couples where MD-TESE is involved. The first and probably most common policy is to perform a diagnostic MD-TESE combined with cryopreservation of testicular tissue in the case of sperm retrieval (20). No parallel hormonal treatment and oocyte retrieval of the female partner end in less emotional and financial implications when sperm recovery is unsuccessful. However, in severe cases of NOA, only occasional spermatozoa are found after dissection of tubules. In subsequent IVF cycles, a high risk of not finding sufficient sperm after thawing has been reported (17).

Another option is to cryopreserve oocytes prior to MD-TESE. If sperm are retrieved after MD-TESE, oocytes can be thawed and microinjected with testicular sperm. In case no sperm are retrieved, oocytes might remain cryopreserved and subsequent treatment with donor sperm is possible if accepted by the couple. However, oocyte freezing prior to MD-TESE has financial implications, and the optimal number of vitrified oocytes recommended prior to MD-TESE has been a matter of debate (21). The third alternative is to run a parallel IVF cycle along with the MD-TESE procedure (10). Sperm retrieval is performed in the morning and oocyte recovery later on the same day. Fertilizing retrieved fresh oocytes with prepared donor sperm on the same day as MD-TESE is also an alternative in cases of no sperm retrieval. However, an ethical consideration arises, in not letting the couple know if sperm from the male partner or a donor will be used for fertilization on the day of MD-TESE and oocyte retrieval.

## Conclusion

In conclusion, there is evidence to suggest that MD-TESE may improve sperm retrieval in men with NOA, but good-quality randomized studies are still lacking. Complications from MD-TESE are rare (<10%) and usually minor (18). The efficacy of MD-TESE with high SRR is due to the new ‘non-blind’ technique making it possible to find tubules with spermatogenesis under an operative microscope. On the other hand, MD-TESE procedures are more time-consuming and require the purchase of an operating microscope. To date, MD-TESE seems to be the best method for the intraoperative identification for sperm-producing tubules in NOA men, and hopefully, the procedure can be offered to more patients with NOA, especially in the Nordic countries where only a few centres have the clinical experience of the technique. However, we do not necessarily recommend MD-TESE to be the only method of sperm retrieval in men with NOA. Up to one-third of patients with NOA will, at a lower cost, achieve sperm recovery by less complicated and invasive needle aspiration and percutaneous biopsy techniques.

## Supplementary Material

Supplemental Material - Etiologies of azoospermia /aspermia Figure 1

Supplemental Material 2b

Supplemental Material 2a

Supplemental Material 1a - 1e
