# Supplementary figures and images for: Utility of micro-TESE in the most severe cases of non-obstructive azoospermia
Source: Ups J Med Sci. 2020 Apr 1;125(2):99–103. doi: 10.1080/03009734.2020.1737600 (PMC7721032; doi:10.1080/03009734.2020.1737600)

# Etiologies of azoospermia /aspermia

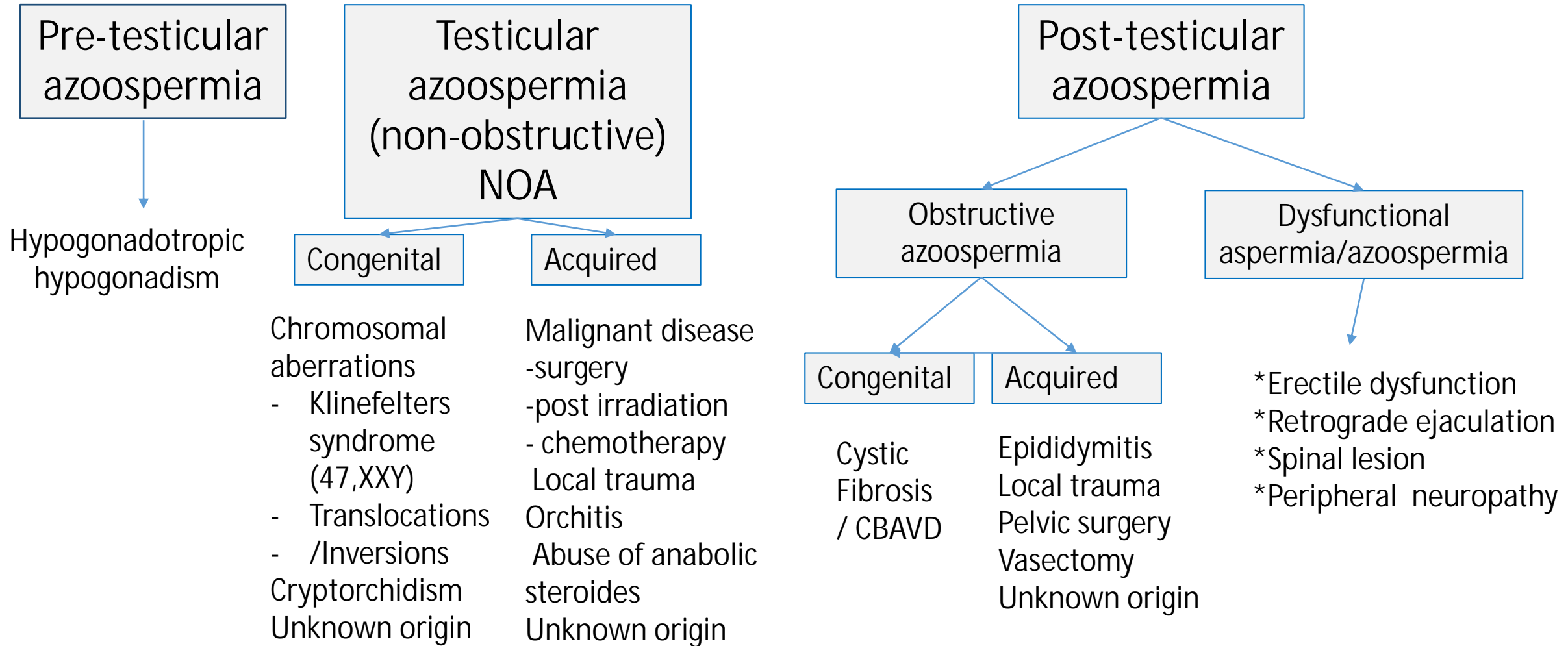

Supplement: Supplemental Material - Etiologies of azoospermia /aspermia Figure 1 [file IUPS_A_1737600_SM8999.pdf]

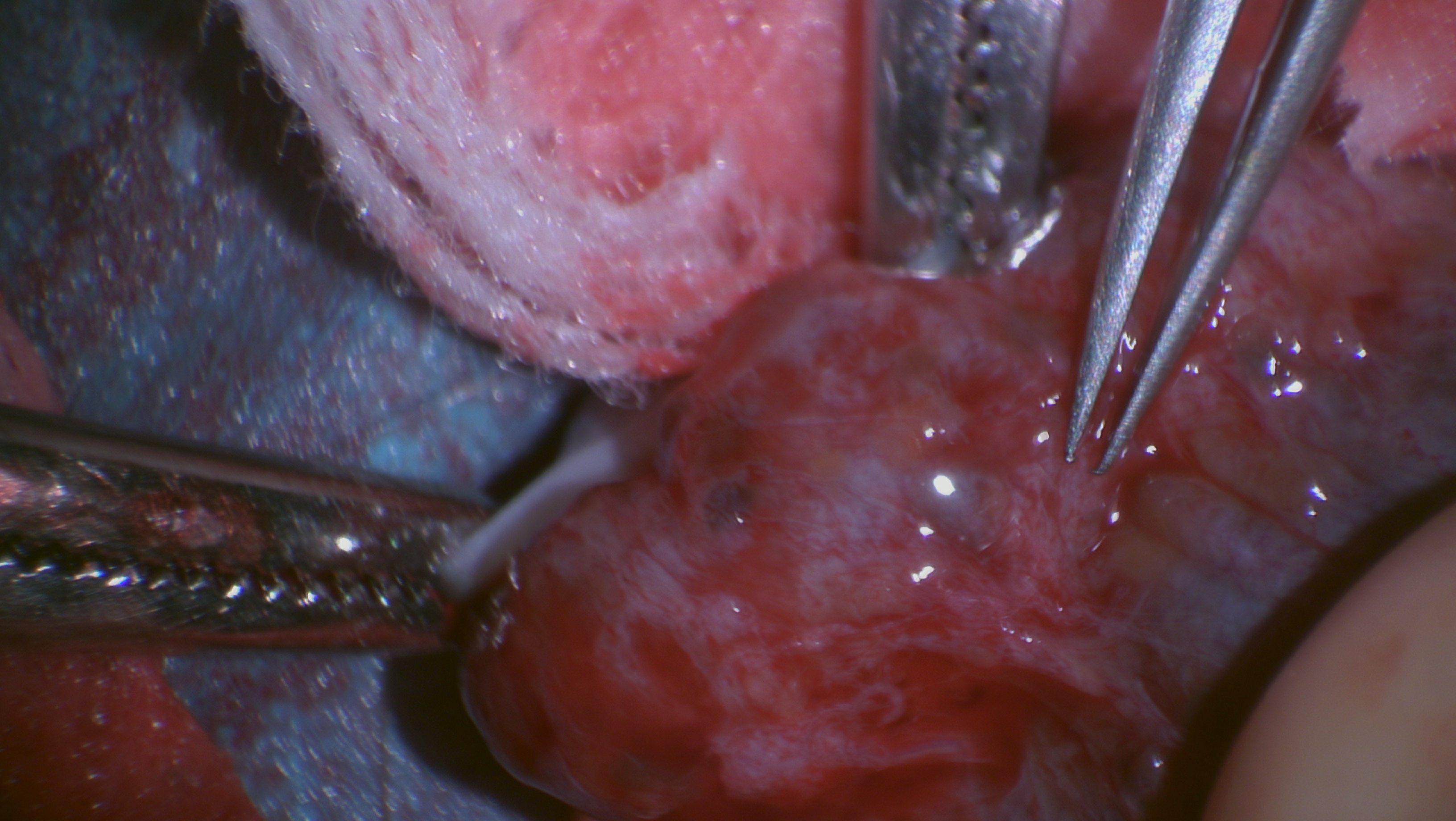

Supplement: Supplemental Material 2b [file IUPS_A_1737600_SM8991.jpg]

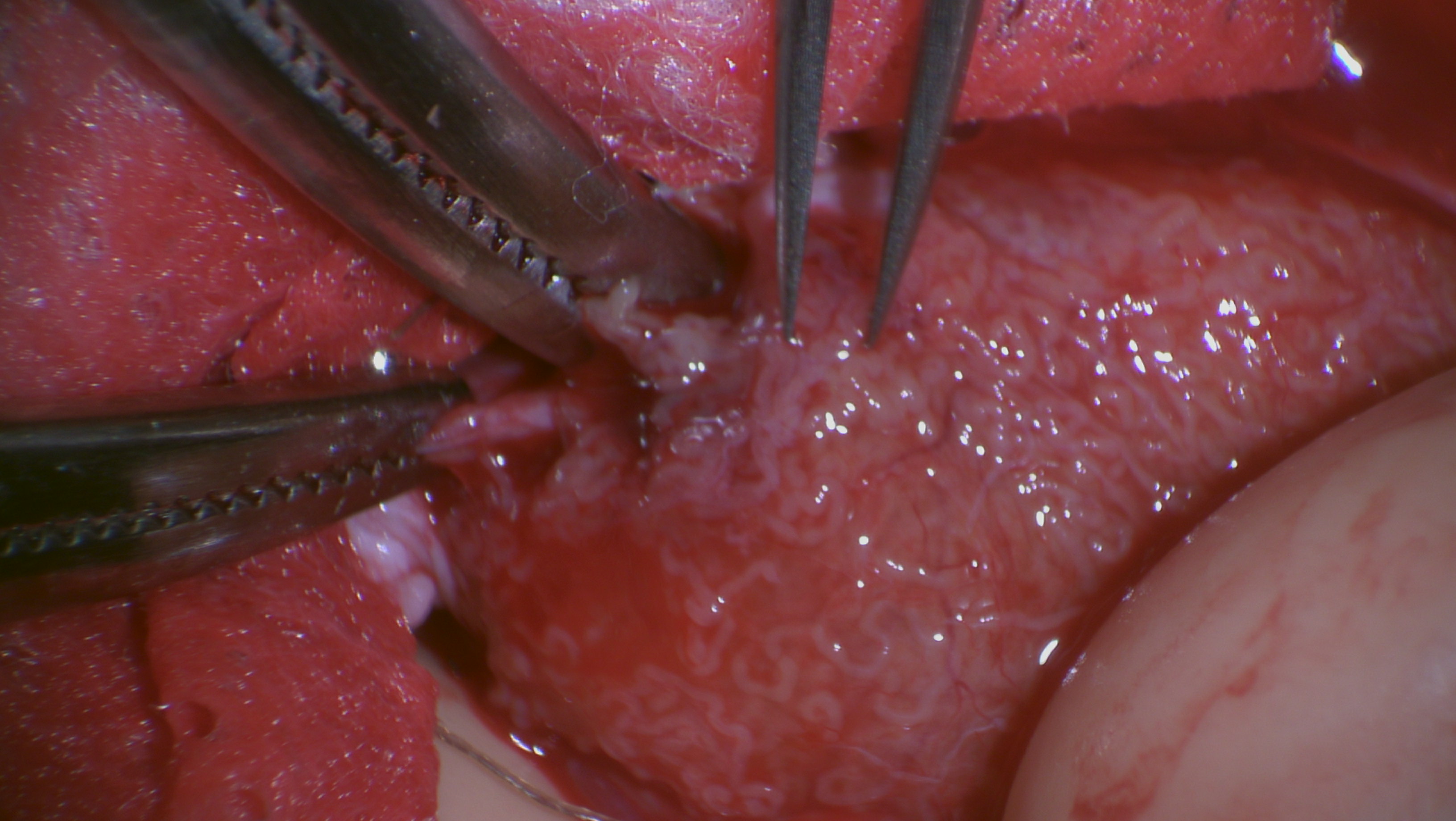

Supplement: Supplemental Material 2a [file IUPS_A_1737600_SM8985.jpg]
