# Supplementary material for: Utility of micro-TESE in the most severe cases of non-obstructive azoospermia
Source: Ups J Med Sci. 2020 Apr 1;125(2):99–103. doi: 10.1080/03009734.2020.1737600 (PMC7721032; doi:10.1080/03009734.2020.1737600)
Supplement: Supplemental Material 1a - 1e [file IUPS_A_1737600_SM8972.pptx]

## Slide 1
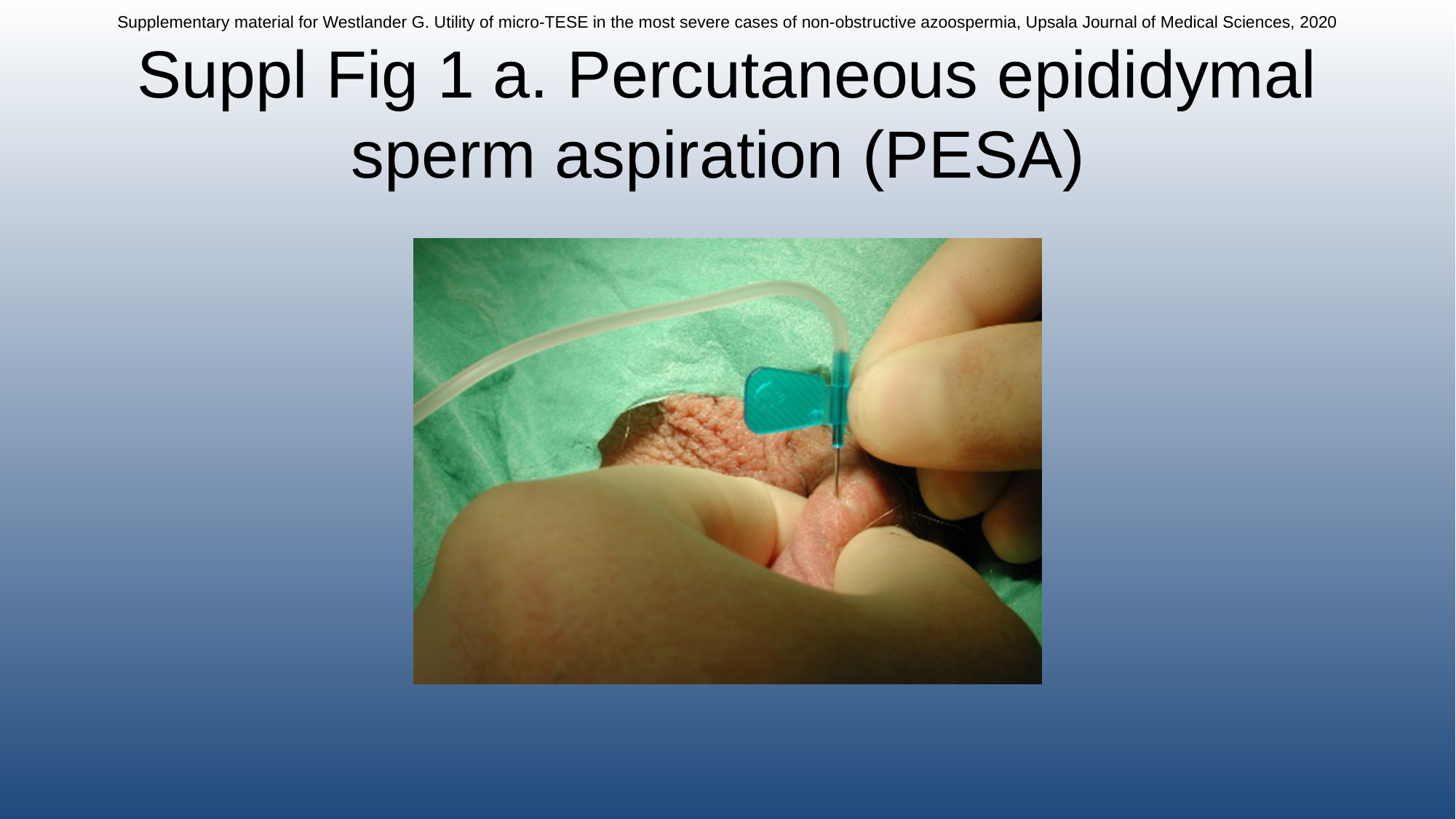

# Supplementary material for Westlander G. Utility of micro-TESE in the most severe cases of non-obstructive azoospermia, Upsala Journal of Medical Sciences, 2020 Suppl Fig 1 a. Percutaneous epididymal sperm aspiration (PESA)

## Slide 2
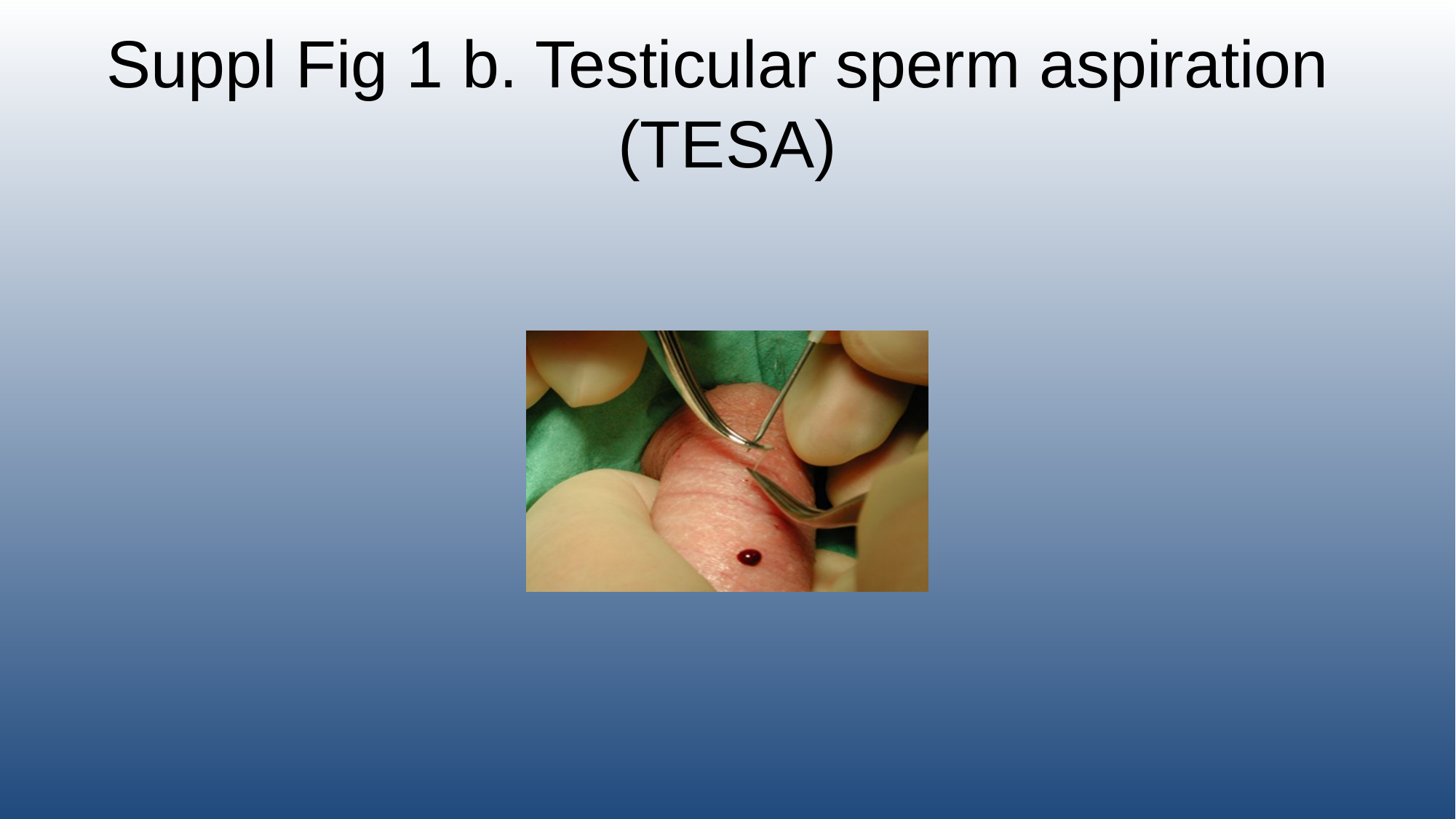

# Suppl Fig 1 b. Testicular sperm aspiration (TESA)

## Slide 3
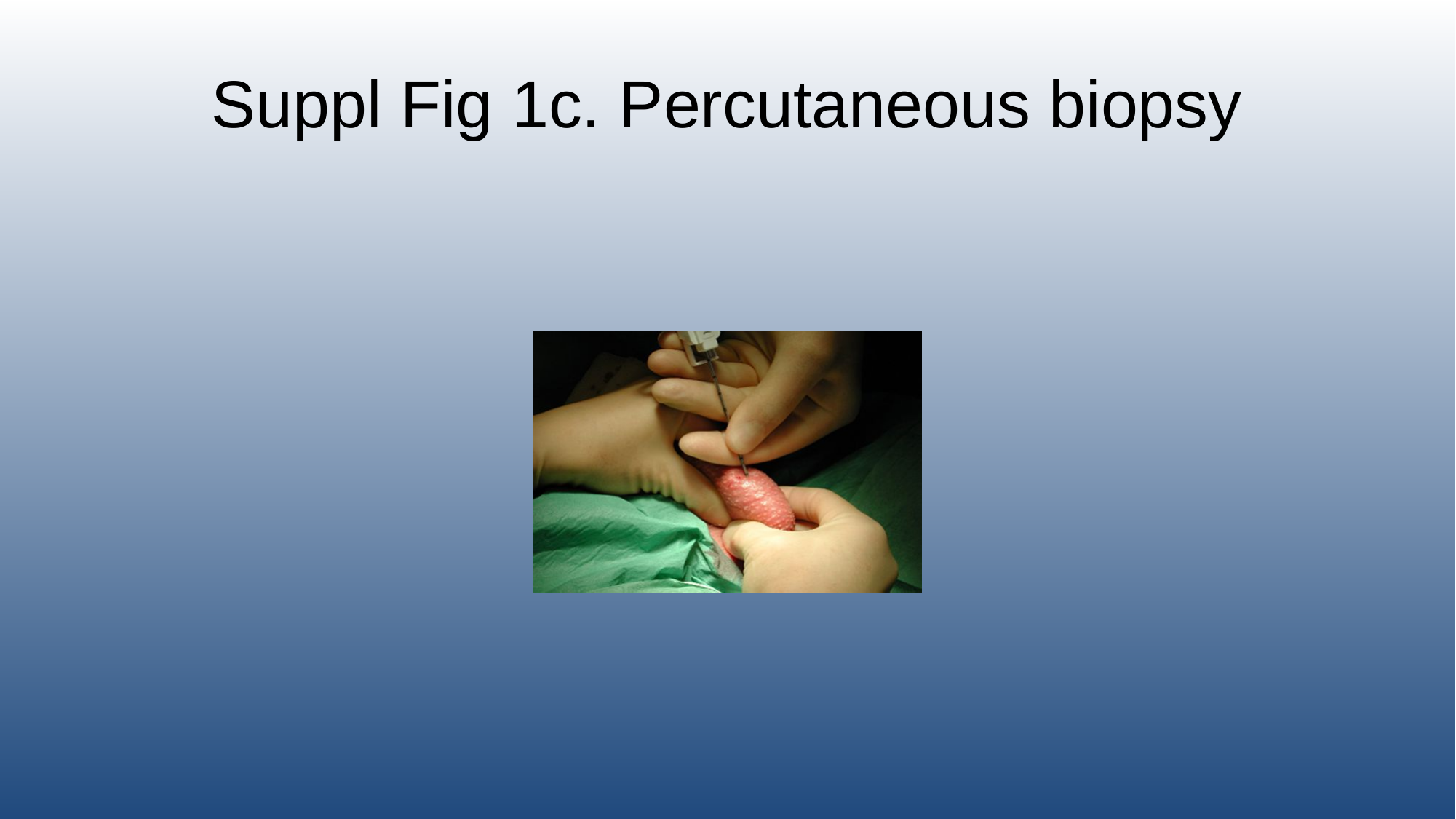

# Suppl Fig 1c. Percutaneous biopsy

## Slide 4
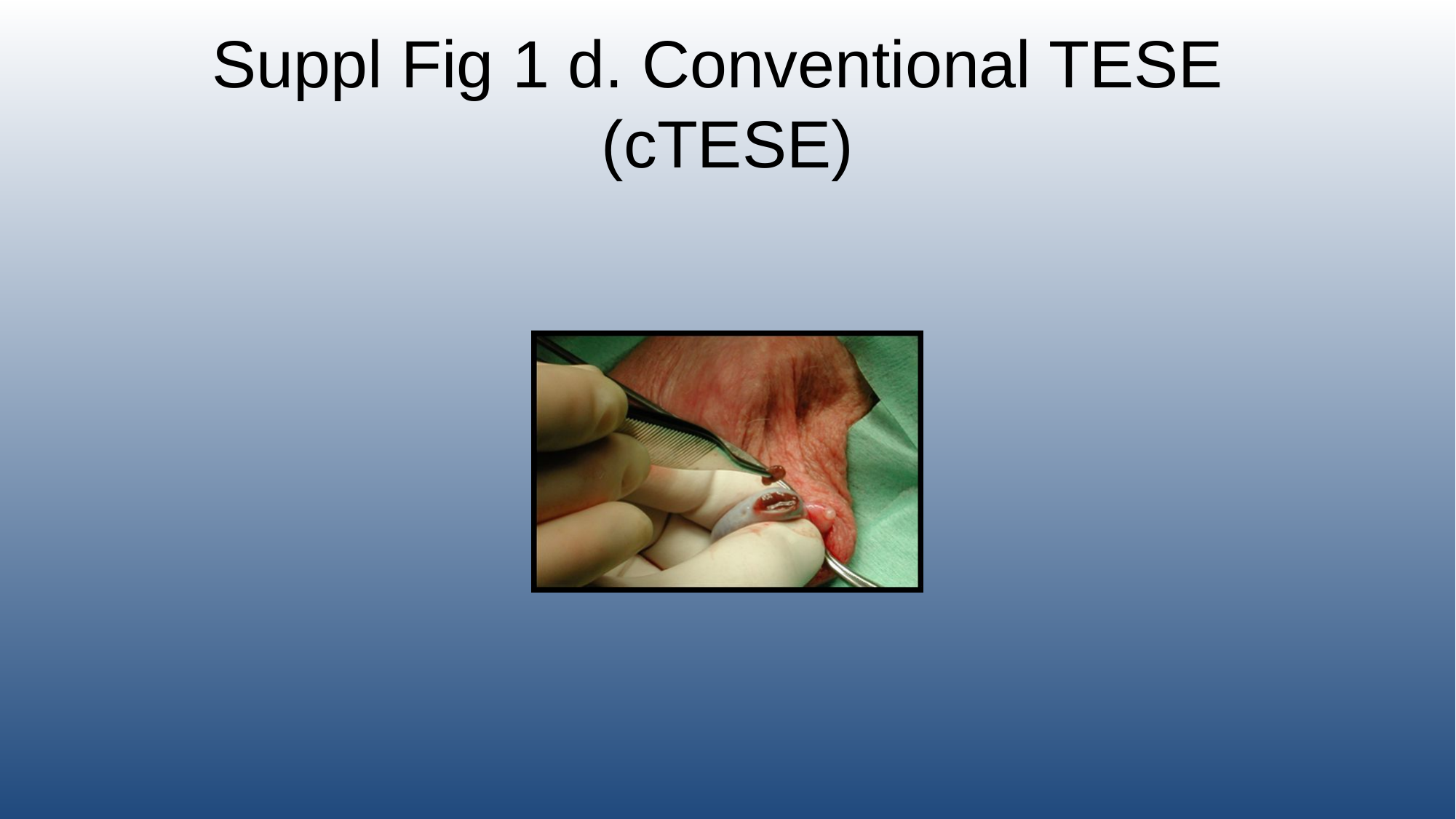

# Suppl Fig 1 d. Conventional TESE (cTESE)

## Slide 5
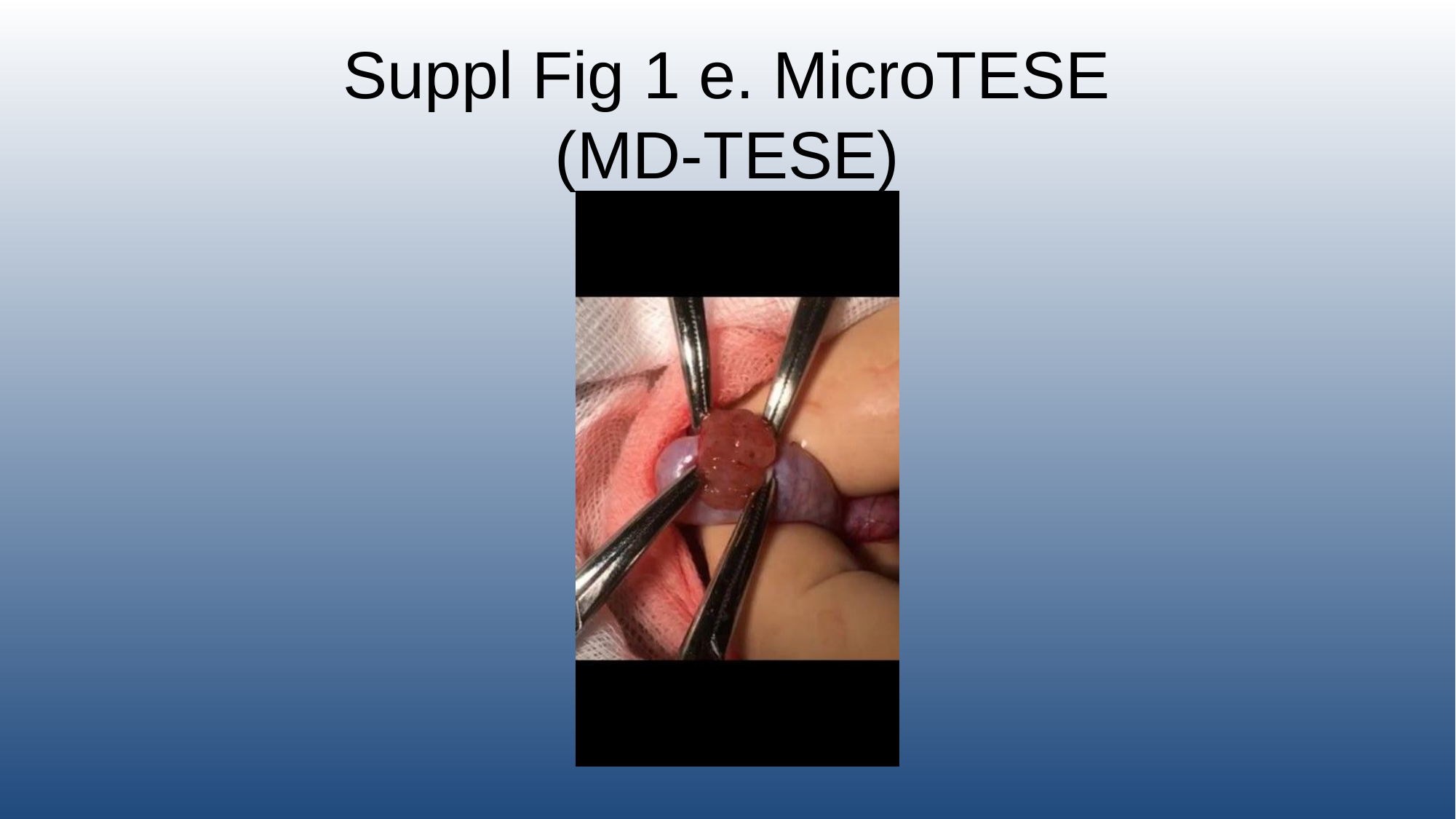

# Suppl Fig 1 e. MicroTESE (MD-TESE)
